# Lippia sidoides essential oil at concentration of 0.25% provided improvements in microbiota and intestine integrity of Danio rerio

**DOI:** 10.29374/2527-2179.bjvm005323

**Published:** 2024-02-14

**Authors:** Lucas Cardoso, Marco Shizuo Owatari, Francisco Célio Maia Chaves, Tamiris Henrique Ferreira, Domickson Silva Costa, William Eduardo Furtado, Marília Tedesco, Luciana Aparecida Honorato, José Luiz Pedreira Mouriño, Maurício Laterça Martins

**Affiliations:** 1 Aquaculture engineer. DSc., Aquatic Organisms Health Laboratory (AQUOS), Departamento de Aquicultura, Universidade Federal de Santa Catarina (UFSC), Florianópolis, SC, Brazil.; 2 Agronomist, DSc;. Embrapa Amazônia Ocidental, Manaus, AM, Brazil.; 3 Aquaculture engineer, DSc., Departamento de Doenças Infecciosas e Saúde Pública, Colégio do Jockey Club de Medicina Veterinária e Ciências da Vida, Universidade da Cidade de Hong Kong, Hong Kong, China.; 4 Veterinarian. DSc., UFSC, Florianópolis SC, Brazil.

**Keywords:** aquaculture, dietary supplementation, microbiome, zebrafish, aquicultura, suplementação dietética, microbioma, peixe-zebra

## Abstract

The study evaluated the effects of dietary supplementation with *Lippia sidoides* essential oil on the microbiota and intestinal morphology of *Danio rerio*. For this, 448 fish were randomly distributed in 28 tanks divided into a control group fed a commercial diet without supplementation, a group fed a commercial diet containing grain alcohol and five groups fed a commercial diet containing essential oil of *L. sidoides* (LSEO) at concentrations of 0.25%, 0.50%, 0.75%, 1.00% and 1.25%. After the period of dietary supplementation, biological materials were collected for microbiological and histological analyses. There were no significant differences regarding the microbiological count between the groups. Diversity of the microbiome was higher in 0.25% group than in control group. LSEO inhibited the growth of potentially pathogenic bacteria. Fish fed LSEO_0.25%_ showed greater intestinal histomorphometric indices. The inclusion of LSEO at 0.25% in the diet of *D. rerio* provided improvements in fish microbiota and intestine integrity.

## Resumo

O estudo avaliou os efeitos da suplementação dietária com óleo essencial de *Lippia sidoides* sobre a microbiota e a morfologia intestinal de *Danio rerio*. Para isso, 448 peixes foram distribuídos aleatoriamente em 28 tanques divididos em grupo controle alimentado com dieta comercial sem suplementação, grupo alimentado com dieta comercial contendo álcool de cereais e cinco grupos alimentados com dieta comercial contendo óleo essencial de *L. sidoides* (LSEO) em concentrações de 0,25%, 0,50%, 0,75%, 1,00% e 1,25%. Após o período de suplementação alimentar, foram coletadas amostras biológicas para análises microbiológicas e histológicas. Não houve diferenças significativas quanto à contagem microbiológica entre os grupos. A diversidade do microbioma foi maior no grupo de 0,25% do que no grupo controle. LSEO inibiu o crescimento de bactérias potencialmente patogênicas. Peixes alimentados com LSEO 0,25% apresentaram maiores índices histomorfométricos intestinais. A inclusão de LSEO a 0,25% na dieta de *D. rerio* proporcionou melhorias na microbiota e na integridade intestinal dos peixes.

## Introduction

The aquatic environment can influence the composition of the intestinal microflora of fish and reveal information about the microbiological conditions of the water (Kord et al., 2022; Romero et al., 2014; Yoshimizu & Kimura, 1976). However, feed intake plays a key role in this equation, favouring the composition of the intestinal microbiota of animals (Fetissov, 2017). The abundance and diversity of nutrients ingested through the diet makes the intestine a favourable environment for bacterial colonization (Rohani et al., 2022).

The intestinal microbiota of fish acts in the proper development and maturation of the gastrointestinal tract, nutritional, physiological, and immune functions (Islam et al., 2021). However, the microbial composition of intestine can change due to the ingestion of functional foods such as essential oils from plants (Carda-Diéguez et al., 2014). Such changes in the intestinal microbiota may be related to the lower susceptibility of beneficial bacteria to the antimicrobial effects of essential oils, selectively stimulating the proliferation of beneficial bacteria and reducing the number of potentially pathogenic bacteria in the intestine (He et al., 2017).

Essential oils can strengthen the immune system and prevent disease (Hassan et al., 2014), as well as act on the cellular structures of bacteria by modifying the lipid composition and fluidity of the cell membrane (Lambert et al., 2001), inhibiting bacterial growth (Cunha et al., 2018).

Species of the *Lippia* genus are rich in aromatic essential oil and have been explored in several fields, such as veterinary medicine, microbiology, parasitology, zootechnics, and aquaculture, owing to their bioactive potential and ease of use on an industrial scale (Soares & Tavares-Dias, 2013). *Lippia sidoides* (pepper rosemary) stand out within the genus, whose essential oil has antibacterial, antiparasitic, and antioxidant effects, which are strongly associated with the presence of its primary compound, thymol (Bakkali et al., 2008).

Essential oils are considered as environmentally friendly additives as they cause less impact on the aquatic environment and, consequently, on fish (Dawood et al., 2022). Over the years, zebrafish (*Danio rerio*) have proven to be a practical biological model of excellent quality due to some characteristics intrinsic to the species (Sanden et al., 2012). *D. rerio* is a small freshwater teleost fish from tropical regions and is easily cultivated in small aquariums due to its minimal space requirements. It has high fecundity (200 eggs per couple) and has a low cost to be cultivated (Martins et al., 2017).

In recent years, *D. rerio* has aroused the interest of the scientific community and has been used as a biological model for numerous studies aimed at a better understanding of aquatic pollution (Mauro et al., 2021; Porretti et al., 2022) and diseases humans (Howe et al., 2013). Furthermore, studies using *D. rerio* as a biological model can serve as a database for other fish species. For this reason, the aim of this study was to evaluate the effects of dietary supplementation of *L. sidoides* essential oil on the microbiota and intestinal histomorphology of *D. rerio*.

## Materials and methods

### Essential oil extraction and chemical composition analysis

The essential oil was obtained from EMBRAPA Amazônia Ocidental, located in Manaus, AM (03° 06′23.04″ S and 60° 01′35.14″ W), with average altitude of 50 m and average annual rainfall of 2200 mm. The plants were collected in the morning and the material was processed at the Laboratory of Medicinal Plants and Phytochemistry of Embrapa Amazônia Ocidental, Manaus, Brazil. Oil extraction was carried out by the hydrodistillation method, using a Clevenger-type equipment. After that, the oils were kept refrigerated at -18 °C in dark glasses. For chemical composition analysis, an Agilent (Palo Alto, USA) 7890A gas chromatograph equipped with an HP-5 capillary column (5%-diphenyl-95%-dimethyl silicon) was used. The temperature was programmed at 60 to 240 °C, at 3 °C min^-1^ and hydrogen was used as carrier gas (1.5 mL min^-1^). A total of 1.0 μL of 1% EO solution in dichloromethane (Merck Millipore, Darmstadt, Germany) with flow division (1:100, inlet at 250 °C) was injected.

The mass spectrum was obtained on an Agilent 5973 N system operated in electronic impact mode (EIMS) at 70 *e*V, coupled to an Agilent 6890 chromatograph using the same injection and temperature procedure mentioned above. Retention indices were calculated from the retention times of compounds of a series of n-alkanes (C7-C26). The identification and quantification of the major compounds were performed by comparing the mass spectrum obtained with data from the spectral library (Wiley 6th Ed.) and by the retention indices calculated and compared with published values ​​(Adams, 2007). The species was deposited at the EAFM Herbarium (Federal Institute of Education, Science and Technology of Amazonas - CMZL) under number 13,882: scientific name: *Lippia sidoides*; common name: pepper rosemary; family: Verbenaceae. *L. sidoides* essential oil showed thymol as the major component (72.2%), followed by ρ-cymene (8.1%), and (E)-caryophyllene (4.9%). Less present compounds were: aromadendrene (0.4%); α-pinene, viridiflorene and δ-cadinene (0.3% each); α-humulene and spathulenol (0.2% each); β-pinene, α-phellandrene, δ-3-carene, (Z)-β-ocimene, (E)-β-ocimene, terpinolene, linalool, α-terpineol, not identified; allo-aromadendrene and α-muurolene (0, 1% each).

### Experimental diet

The experimental diets were based on the extruded commercial feed *Nutripiscis Starter-45* 0.8 mm (Crude protein 45%, ether extract 9%, minerals 15% and crude fiber 4%) from *Neovia Nutrição e Saúde Animal Ltda,* and the inclusion of LSEO (essential oil + cereal alcohol) in the feed was carried out by the conventional spraying method with manual sprayer. Grain alcohol was used as an incorporation vehicle for oil dilution. For each 100 g of feed, 10 mL of grain alcohol was sprinkled with *L. sidoides* oil diluted in different concentrations. In the grain alcohol treatment (GA), there was only grain alcohol spraying on the feed in the same proportion (Dairiki et al., 2013).

The feed was dried at room temperature for 24 h and then stored at -18°C. The day before supply, the feed was weighed and stored at 4 °C until feeding. The experimental diets were offered for a period of 15 days, three times daily with an amount corresponding to 5% of the fish total biomass.

### Experimental design

A total of 448 fish (0.3±0.04g weight and 2.7±0.23 cm length) were distributed in 28 rectangular plastic aquariums with a useful volume of 16L (n=16), totalizing seven groups in quadruplicates. Treatments consisted of a control group fed a commercial diet without supplementation, a group fed a commercial diet containing grain alcohol, and five groups fed a commercial diet containing *L. sidoides* essential oil (LSEO) at concentrations of 0.25%, 0.50%, 0.75%, 1.00% and 1.25%.

The experimental units were coupled to a water recirculation system with mechanical and biological filters according to Owatari et al. (2018), and ultraviolet sterilization. The water turnover rate was approximately 20% per day, and the excess feces was siphoned off twice daily. The photoperiod was set at 12 h light. Water temperature, pH, and dissolved oxygen (DO) were measured daily with a multiparameter (Hanna®, model HI-9828, USA), and total ammonia, nitrite, and nitrate were also measured daily but with a commercial colorimetric kit (Alfakit^®^, Brazil). During the experimental period, water quality variables remained within the comfort range for *D. rerio*. The average temperature was 27.40 ± 2.60 ºC, DO 7.33 ± 1.00 mg L^-1^, pH 6.62 ± 0.21, total ammonia 0.06 ± 0 .02 mg L^-1^, nitrite 0.02 ± 0.02 mg L^-1^, and nitrate 0.00 ± 0.00 mg L^-1^.

At the end of the 15-day dietary supplementation period, the animals were anesthetized (60 µL of clove oil stock solution (10%, diluted in ethanol) per 1 mL of water) (Grush et al., 2004) and euthanized for collection of biological material.

### Bacteriological count of the intestinal tract

At the end of the dietary supplementation period, the fish were fasted for 24 h and the intestines of four animals per aquarium were aseptically collected, weighed, macerated, serially diluted in 0.65% sterile saline solution at a ratio of 1:10, and then dilutions from 10^-4^ to 10^-9^ were seeded in: Man Rogosa Sharpe agar medium (MRS, Himedia^®^ Mumbai, India) for lactic acid bacteria cultivable totals, with aniline blue and Tryptone Soy Agar (TSA, Himedia^®^ Mumbai, India) for cultivable total heterotrophic bacteria. Dilutions 10^-1^ to 10^-4^ were seeded on: Thiosulfate Citrate Bile Sucrose agar (TCBS, Himedia® Mumbai, India) for cultivable vibrionaceae and Cetrimid agar (Himedia^®^ Mumbai, India) for cultivable *Pseudomonas* sp. All culture media were incubated at 30 ºC for 24 h, apart from MRS, which was incubated at 35 ºC for 48 h.

### Analysis of richness, diversity, and abundance of the intestinal microbiota

To verify the changes in the intestinal microbiota at the species level, the Next-generation sequencing (NGS) analysis was performed using MiSeq System (Illumina, Inc.). For this, the intestinal tract of two animals per aquarium was removed aseptically, had its contents scraped with the aid of a swab and stored in a microtube containing NeoSample-Z Solution (Neoprospecta microbiome technologies^®^, Santa Catarina, Brazil) at room temperature. Finally, the samples were sent to Neoprospecta Microbiome Technologies^®^ (Santa Catarina, Brazil) to carry out the analysis of operational taxonomic units (OTU) using metagenomics, a technique capable of providing data on the relative abundance, richness and diversity of bacteria present in the microbiota gut at the species level.

### Histological analysis

Intestine fragments from 84 fish (3 per aquarium) were collected and fixed in a 10% buffered formalin solution. Organs were dehydrated in an increasing series of alcohol, clarified in xylene, embedded in paraffin, and ultrathin sliced (4 μm thickness) (micro-tome PAT-54 MR10) (Humason, 1962). Permanent slides were prepared in Entellan^®^ media and analysed under differential interference contrast (DIC) microscopy (ZEISS, Axio Imager A.2, Gottingen, Germany). The histological changes in intestinal histomorphometry were performed by measuring the length, width, area, perimeter of the villi, and quantifying the goblet cells per villi.

### Statistical analysis

All data were submitted to the Shapiro-Wilk and Levene tests to verify normality and homoscedasticity of variance, respectively. Non-homogeneous data were transformed into log_10_ (x +1). Once the assumptions were guaranteed, the data were submitted to one-way ANOVA analysis of variance and the means were compared by Tukey's test. All tests were performed at a significance level of 5% using the Statistica 10.0 software. The QIIME program (Quantitative Insights Into Microbial Ecology) was used to identify the core microbiota, defined in this study as OTU, which were related to their corresponding taxa. The results of the microbiome characterization were presented at taxonomic levels. Heatmap plots for phylum, class, and genus were produced from Heatmapper (Babicki et al., 2016). For this, the OTU were grouped (clustering) by the average linkage method (average linkage) and Euclidean was used for the distance (Yang & Xu, 2020). As for the Venn diagram, the InteractiVenn was used (Heberle et al., 2015). Furthermore, the diversity profile (Diversity Profiles) and Principal Coordinate Analysis (PCoA) were performed using the PAST 4.03 program (Hammer et al., 2001). For PCoA, the similarity index was calculated by Euclidean.

## Results

**Figure 1:**
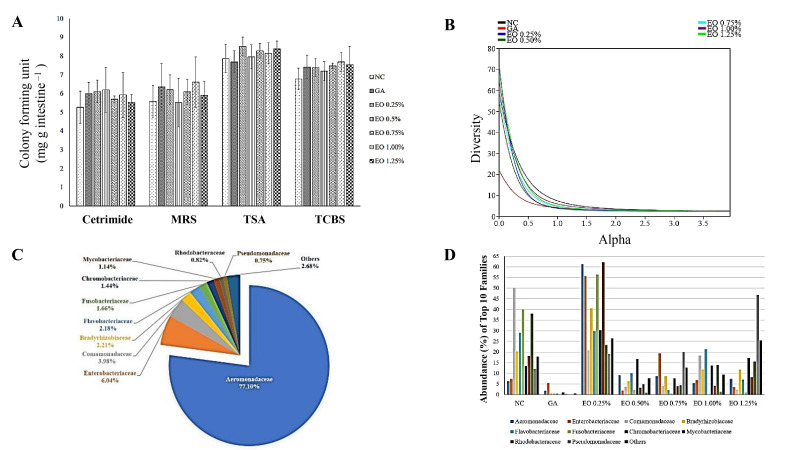
Microbiological Analysis of *Danio rerio* after 15 days of feeding with diet supplemented with *Lippia sidoides* essential oil in concentrations of 0.25%, 0.50%, 0.75%, 1.00%, and 1.25%, or not supplemented negative control (NC) and supplemented with grain alcohol (GA). In (A) Microbiological count of *Pseudomonas* sp. (Cetrimide), total lactic acid bacteria (MRS), total heterotrophic bacteria (TSA) and Vibrionacea (TCBS) per gram of intestine. The data are presented as mean and standard deviation. In (B) Alpha Diversity profile of microbial species in the intestinal tract. In (C) Relative abundance (%) of the top 10 Families most found in the intestinal microbiota of *D. rerio* regardless of the experimental group. In (D) Relative Abundance (%) of the top 10 Families according to the experimental groups.

In the microbiological analyses of the intestinal tract, no significant differences were found between animals fed a supplemented diet and those fed a diet without LSEO supplementation (Figure 1A).

Regarding the diversity index of the microbial community of the animals in the present study, it was possible to observe that the animals of the 0.25% treatment presented higher values ​​of Rate (71), OTUs (68099), Dominance (0.51) and Berger-Parker Index (0.70), and lower values ​​for Simpson Indexes (0.49), Shannon (1314), Evennes (0.05), Menhinick (0.27), Margalef (6.29), Equitability (0.31) compared to the fish of the other groups; while the Brillouin Index was higher (2162) in 1.00% treatment and lower (2.61) in animals from NC. The Fisher Alpha Index was higher (9498) in 1.00% group and lower (10.06) in 1.25% group, while the Chao-1 index was higher (71) in 0.25% and 1.25% groups (Figure 1B). The most preponderant Families, in terms of Relative Abundance (%), were Aeromonodaceae (77.10%) and Enterobacteriaceae (6.04%). The 0.25% treatment presented more significant values for this index when compared to the other groups (Figure 1C and 1D). In terms of the total number of Operational Taxonomic Units (OTU), the most expressive value (56%) was observed in the animals of the 0.25% group (Table 1).

**Table 1:** Diversity index of the microbial community of the intestinal tract of *Danio rerio* after 15 days of dietary supplementation with *Lippia sidoides* essential oil in concentrations of 0.25%, 0.50%, 0.75%, 1.00%, and 1.25%, or not supplemented negative control (NC) and supplemented with grain alcohol (GA).

Diversity index				Treatments			
	NC	GA	LSEO_0.25%_	LSEO_0.50%_	LSEO_0.75%_	LSEO_1.00%_	LSEO_1.25%_
*Taxa*	58	21	71	54	57	64	69
*OTUs*	12629	2215	68099	10011	10856	8008	9580
*Dominance*	0.11	0.32	0.51	0.38	0.29	0.2	0.34
*Simpson*	0.89	0.68	0.49	0.62	0.71	0.8	0.66
*Shannon*	2622	1451	1314	1465	1754	2179	1826
*Evennes*	0.24	0.2	0.05	0.08	0.1	0.14	0.09
*Brillouin*	2.61	1432	1311	1454	1742	2162	1811
*Menhinick*	0.52	0.45	0.27	0.54	0.55	0.71	0.7
*Margalef*	6036	2596	6.29	5754	6026	7009	7418
*Equitability*	0.64	0.48	0.31	0.37	0.43	0.52	0.43
*Fisher_alpha*	7856	3212	7827	7503	7886	9498	10.06
*Berger-Parker*	0.17	0.46	0.7	0.52	0.42	0.36	0.55
*Chao-1*	59	22	71	55	58	67.5	71

**Figure 2:**
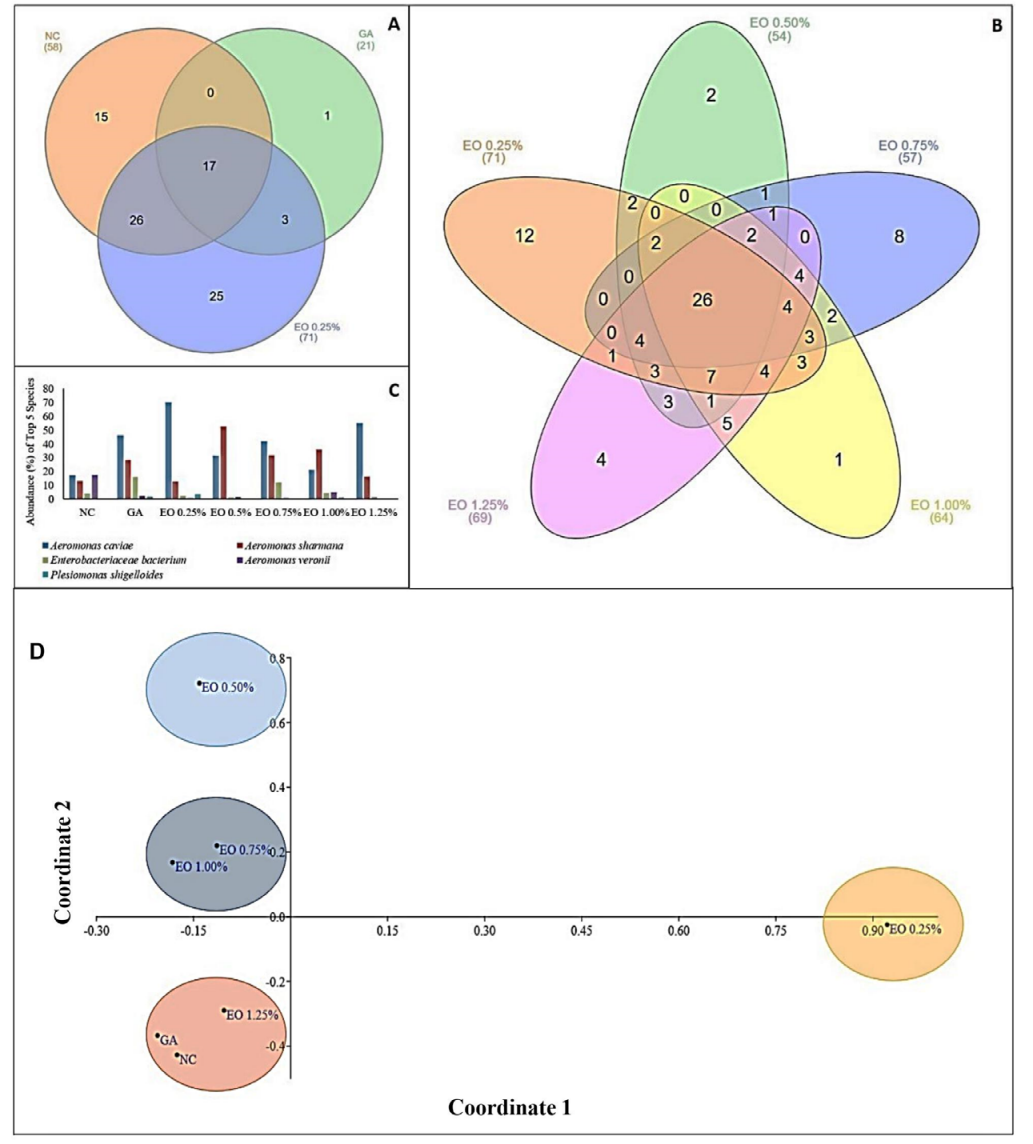
Microbiome Analysis of *Danio rerio* after 15 days of feeding with diet supplemented with *Lippia sidoides* essential oil in concentrations of 0.25%, 0.50%, 0.75%, 1.00%, and 1.25%, or not supplemented negative control (NC) and supplemented with grain alcohol (GA). In (A) and (B) Venn Diagram and Relative Abundance (%) at Species level, singular and shared, of the bacterial microbiota. In (C) Relative abundance (%) of the five most common species. In (D) Principal Coordinate Analysis (PCoA) used to determine the metric distance and ordering of the microbial communities present in the intestinal tract of *D. rerio*.

From the Venn Diagram (Figure 2A and 2B), it was possible to observe that the NC group presented the highest values (41) in terms of singular species, whereas in terms of species shared between the groups, it was observed that 1.25% treatment shares 65 species with the other groups. However, dietary supplementation with LSEO stimulated the emergence of a greater number of unique species (50 OTU) if compared to that found in the intestinal microbiota of fish from the control group (7 OTU).

**Figure 3:**
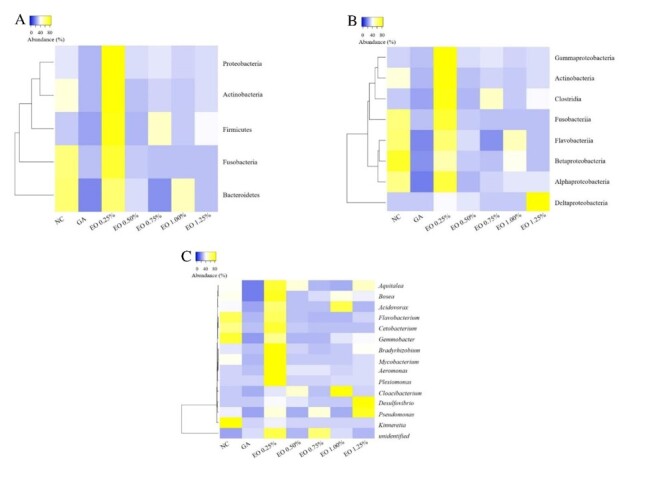
Microbiome Analysis of *Danio rerio* after 15 days of feeding with diet supplemented with *Lippia sidoides* essential oil in concentrations of 0.25%, 0.50%, 0.75%, 1.00%, and 1.25%, or not supplemented negative control (NC) and supplemented with grain alcohol (GA). In (A**)** Heat map of Relative Abundance (%) at Phylum level, grouped by the average linkage method (average linkage) and distanced by Euclidean. In (B) Heatmap of Relative Abundances (%) at Class level, clustered by the average linkage method (average linkage) and distanced by Euclidean. In (C) Heat map of the Relative Abundance (%) of the 15 most found genera, grouped by the average linkage method (average linkage) and distanced by Euclidean.

Regarding relative abundance (%), the five most preponderant species were *Aeromonas caviae*, *A. veronii*, *A. sharmana*, *Enterobacteriaceae bacterium* and *Plesiomonas shigelloides* (Figure 2C). Principal coordinate analysis (PCoA) showed that the composition of the intestinal microbiota of fish in the 0.25% group was considerably different from that of the other groups (Figure 2D). The highest values of relative abundance (%) in terms of Phyla and Classes were observed in the 0.25% treatment (Figure 3A and 3B). The Relative Abundance (%) of the 15 main genera was higher in the 0.25% group than in the other groups (Figure 3C).

## Discussion

Essential oils have achieved a prominent position worldwide owing to scientific advances in recent decades, which have directly contributed to increasing their applicability in different fields of health, functional nutrition and the resistance of aquatic animals against infectious diseases (Dawood et al., 2022). According to Dawood et al. (2020), essential oils chemical constituents cause positive effects on the intestinal health of animals by favoring the proliferation of beneficial microorganisms and reducing the abundance of pathogenic microorganisms. However, changes in the intestinal microbiota may be related, among other factors, to the duration of dietary supplementation (Zhang et al., 2020). Despite this, in the present study it was possible to observe significant improvements in the microbiome in just 15 days of supplementation.

Similarly, He et al. (2017) reported a significant increase in species richness and diversity in the intestinal microbiota of *Litopenaeus vannamei* fed a diet containing organic acid and essential oil. Despite this, Huyben et al. (2021) did not observe significant differences between the OTU of rainbow trout (*Oncorhynchus mykiss*) supplemented with a diet containing essential oil and the control group. However, they suggested that the organic acid and essential oil increased midgut health by regulating the microbial community. On the other hand, in the present study, the reduction in bacterial species richness values ​​in the intestinal tract of fish fed a diet containing grain alcohol is a result that agrees with that reported by Silva et al. (2012), who found propolis to be more effective against *Staphylococcus* spp. when this compound was extracted with the solvent grain alcohol. As this type of alcohol is an ethanol commonly used in the food, pharmaceutical and cosmetic industries, and can act in the denaturation of proteins and in the dissolution of lipids (Batey, 2017), it possibly led to a decrease in the richness of bacteria in the intestinal microbiota of the fish in this treatment compared to the other groups.

In the present study, the profile of the bacterial community in the intestinal microbiota of *D. rerio* fed with 0.25% differed considerably from the other groups. Likewise Ran et al. (2016) reported that gut microbial community diversity indices were higher in juvenile hybrid tilapia (*Oreochromis niloticus* x *O. aureus*) that received a six-week dietary supplementation with essential oil containing equivalent concentrations of thymol and carvacrol (200 mg Kg^-1^). At the time, the authors found a greater dominance of the Phylum Proteobacteria and the Genus *Plesiomonas* and a lower relative abundance of Bacteroides *Cardinium* and *Leptospirillum* in the treated group.

On the other hand, Reda et al. (2022) found that addition of rice protein concentrate (RPC) reduced microbial diversity regardless of RPC content and that RPC-enriched diets resulted in greater relative abundance of Bacteroidetes and Fusobacteria in the intestine of *Oreochromis niloticus* compared to the intestine of the control fish. However, replacing more than 25% fishmeal with RPC can have deleterious effects on fish. According to Carda-Diéguez et al. (2014), it is possible that the short-term administration of diets containing essential oil causes transient changes in the intestinal microbiota, suggesting that the difference between the diversity of allochthonous and autochthonous bacteria may be linked to fecal content, or rather, to the number of bacteria associated with mucus; in their case, the Bradyrhizobiaceae Family (Class Alphaproteobacteria) was the predominant bacterial group in *Dicentrarchus labrax*. Thus, it is possible that in the present work, the concentration of 0.25% improved the composition of the intestinal mucosa, and consequently enabled the development of the intestinal bacterial community of *D. rerio* from this treatment. The increase in the values ​​of diversity and relative abundance of the intestinal microbiota of fish in this group may also be conditioned to the change in the structure of the intestinal microbiota owing to the greater activity of digestive enzymes and the increase in immunological activity values ​​(Zhang et al., 2020).

The essential oil used in the present study probably acted on the bacterial cell membrane, whose permeability depends on the hydrophobicity of the surrounding solutes, its composition, and the partition coefficient (Lambert et al., 2001; Sikkema et al., 1995) and inhibited the growth of some potentially pathogenic species: *Bosea thiooxidans*, *Burkholderia cepacia*, *Cupriavidus pauculus*, *Leucobacter komagatae*, *Mesorhizobium loti*, *Nitrobacter vulgaris*, *Rhizobium mesosinicum*.

In general context, the lower values ​​of relative abundance, at the level of Phylum, Class, Family and Genus, observed in the other treatments may be related to a possible toxic effect for fish. According to Lee et al. (2003), essential oil carries volatile secondary metabolites with low molecular weight and selective property on the microbiota. It is possible that the higher concentrations of LSEO (0.50%, 0.75%, 1.00% and 1.25%) generated a deleterious effect on the intestinal microbiota of *D. rerio*, causing significant damage such as the partial or total elimination of the bacterial community. In this way, the effectiveness and biological activities of essential oil may be related to the composition, concentration and/or chemotype used. Species-specific actions should be used, given that these substances can cause stress in the hosts if the concentrations and chemotypes are not adequate (Souza et al., 2019).

**Figure 4:**
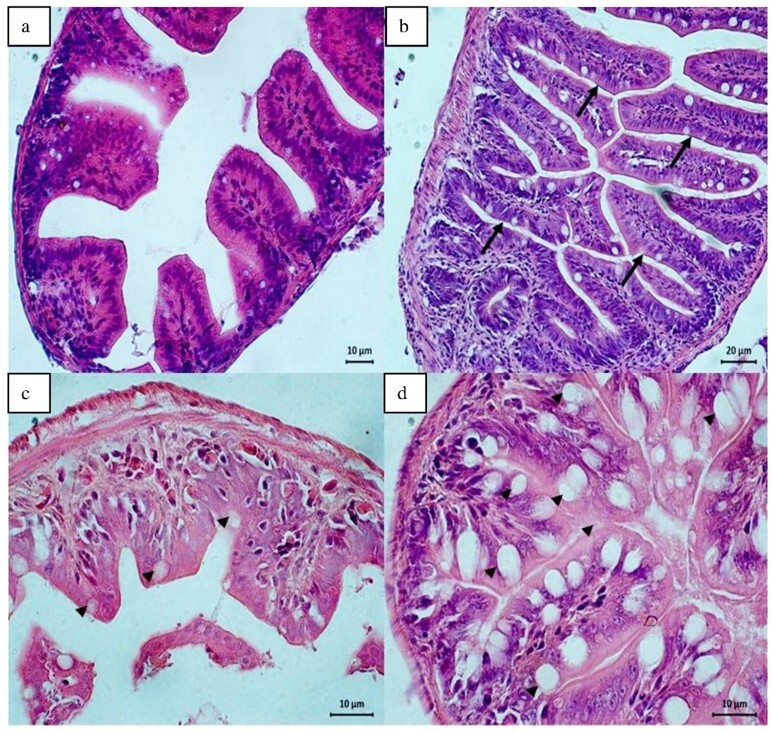
Histological changes in the intestine of *Danio rerio* after 15 days of feeding with diet supplemented with *Lippia sidoides* essential oil in concentrations of 0.25%, 0.50%, 0.75%, 1.00%, and 1.25%, or not supplemented negative control (NC) and supplemented with grain alcohol (GA). In (a), organ with a smaller number of villi in negative control group (arrows). In (b) show the increase in the number, length, and width of villi in 0.25% group (arrowheads). In (c) show the reduced amount of goblet cells in negative control. In (d) there is the presence of villi with a large number of goblet cells in 0.25% group (arrowhead). Hematoxylin and eosin staining. Magnification: scale bars are 10 and 20 μm.

Organs such as the intestine are of vital physiological importance in maintaining fish health (Abdel Rahman et al., 2021). In the intestine, treatment with 0.25% inclusions promoted significant improvements in the increase in the number of villi, villi length, villi width, increase in the number of goblet cells and villi area (Table 2 and Figure 4). The improvement in intestinal morphometric characteristics may be related to the intestinal dysbiosis, i.e., the favoring of beneficial bacteria over harmful bacteria (Rohani et al., 2022). Similarly, Abdel Rahman et al. (2019) found that intestinal villi heights and the number of goblet cells and intraepithelial lymphocytes (IEL) increased significantly in all parts of the intestine of Nile tilapia *Oreochromis niloticus* supplemented with diet containing inclusions of 0.1, 0.2 and 0.4% of Indian Lotus (*Nelumbo nucifera* Gaertn.) compared to the fish of control group. Functional feed additives may have provided energy for the multiplication of beneficial enteric bacteria in the intestine of fish (Robles et al., 2013), as observed for LSEO in *D. rerio* in the present study.

**Table 2:** Histomorphometric changes in the midgut of *Danio rerio* after 15 days dietary supplementation with *Lippia sidoides* essential oil in concentrations of 0.25%, 0.50%, 0.75%, 1.00%, and 1.25%, or not supplemented negative control (NC) and supplemented with grain alcohol (GA). Data presented as mean and standard deviation. Different letters represent a significant difference between the experimental groups by the Tukey test (p <0.05).

*Histomorphometric alterations*	*NC*	*GA*	*LSEO_0.25%_*	*LSEO_0.50%_*	*LSEO_0.75%_*	*LSEO_1.00%_*	*LSEO_1.25%_*	*p value*
*Number of villi*	16.75±5.71^b^	15.92±5.05^bc^	25.08±7.80^a^	10.25±2.30^c^	15.25±4.33^bc^	12.50±2.75^bc^	15.33±3.14^bc^	0.000
*Length (µm)*	65.82±23.39^d^	63.71±26.41^d^	125.00±27.45^a^	121.63±13.22^ab^	95.45±24.46^bc^	93.73±21.73^c^	87.02±6.05^cd^	0.000
*Width (µm)*	38.44±13.97^ab^	34.25±20.21^b^	53.13±13.32^a^	50.18±5.19^ab^	49.47±14.44^ab^	51.27±11.60^a^	39.29±3.82^ab^	0.001
*Number of goblet cells*	66.33±11.42^bc^	71.08±31.69^bc^	124.31±42.02^a^	72.00±14.94^bc^	86.92±28.44^b^	48.25±11.47^c^	69.58±11.33^bc^	0.000
*Area (µm^2^)*	98925.96±39698.67^c^	359795.18±213087.77^ab^	411346.26±187835.02^a^	177558.94±84709.87^c^	245221.14±91069.81^bc^	222321.66±87688.22^bc^	244950.15±78083.48^bc^	0.000
*Perimeter (µm)*	6108.53±2314.45	11104.46±4828.74	240856.81±783667.48	6196.71±2100.62	8427.70±2348.05	7614.76±1943.54	8685.14±2171.60	0.395
*Eosinophilic infiltrate*	31.25±21.65	33.33±22.19	33.33±19,46	35.42±16.71	37.50±27.18	29.17±9.73	33.33±12.31	0.964
*Lymphocytic infiltrate*	16.67±12.31	8.33±12.31	2.08±7.22	10.42±12.87	14.58±19.82	12.50±13.06	14.58±12.87	0.148
*Melanomacrophage*	2.08±7.22	4.17±9.73	0.00±0.00	0.00±0.00	2.08±7.22	0.00±0.00	0.00±0.00	0.350
*Necrosis*	45.83±20.87	31.25±21.65	25.00±21.32	25.00±21.32	39.58±22.51	41.67±24.62	35.42±19.82	0.139
*Vacuolation*	39.58±27.09	33.33±24.62	27.08±22.51	22.92±22.51	39.58±27.09	43.75±21.65	39.58±22.51	0.311

## Conclusion

Therefore, considering the evidence obtained in the present research, we conclude that dietary supplementation with *L. sidoides* essential oil at 0.25% in the feed was beneficial for the fish, modifying the intestinal microbiota, increasing the richness, diversity, and abundance of the microbiome, as well as improving the intestinal morphometric indices, suggesting an optimized modulation of the intestinal microbiota. We recommend further studies with other species of fish or other animal species to investigate the effectiveness of the effects observed here with *L. sidoides* essential oil at a concentration of 0.25%.
